# Learning accurate and interpretable models based on regularized random forests regression

**DOI:** 10.1186/1752-0509-8-S3-S5

**Published:** 2014-10-22

**Authors:** Sheng Liu, Shamitha Dissanayake, Sanjay Patel, Xin Dang, Todd Mlsna, Yixin Chen, Dawn Wilkins

**Affiliations:** 1 Department of Computer and Information Science, University of Mississippi, Weir Hall 201, 38677, University, MS, U.S.A; 2 Department of Chemistry, Mississippi State University, Box 9573, 37962, Mississippi State, MS, U.S.A; 3 Seacoast Science, Inc, 2151 Las Palmas Dr., Suite C, 92011, Carlsbad, CA, U.S.A; 4 Department of Mathematics, University of Mississippi, Hume Hall 305, 38677, University, MS, U.S.A

**Keywords:** Rule based regression, random forests, rule extraction, feature selection, robustness, TCGA

## Abstract

**Background:**

Many biology related research works combine data from multiple sources in an effort to understand the underlying problems. It is important to find and interpret the most important information from these sources. Thus it will be beneficial to have an effective algorithm that can simultaneously extract decision rules and select critical features for good interpretation while preserving the prediction performance.

**Methods:**

In this study, we focus on regression problems for biological data where target outcomes are continuous. In general, models constructed from linear regression approaches are relatively easy to interpret. However, many practical biological applications are nonlinear in essence where we can hardly find a direct linear relationship between input and output. Nonlinear regression techniques can reveal nonlinear relationship of data, but are generally hard for human to interpret. We propose a rule based regression algorithm that uses 1-norm regularized random forests. The proposed approach simultaneously extracts a small number of rules from generated random forests and eliminates unimportant features.

**Results:**

We tested the approach on some biological data sets. The proposed approach is able to construct a significantly smaller set of regression rules using a subset of attributes while achieving prediction performance comparable to that of random forests regression.

**Conclusion:**

It demonstrates high potential in aiding prediction and interpretation of nonlinear relationships of the subject being studied.

## Background

**Figure 1:**
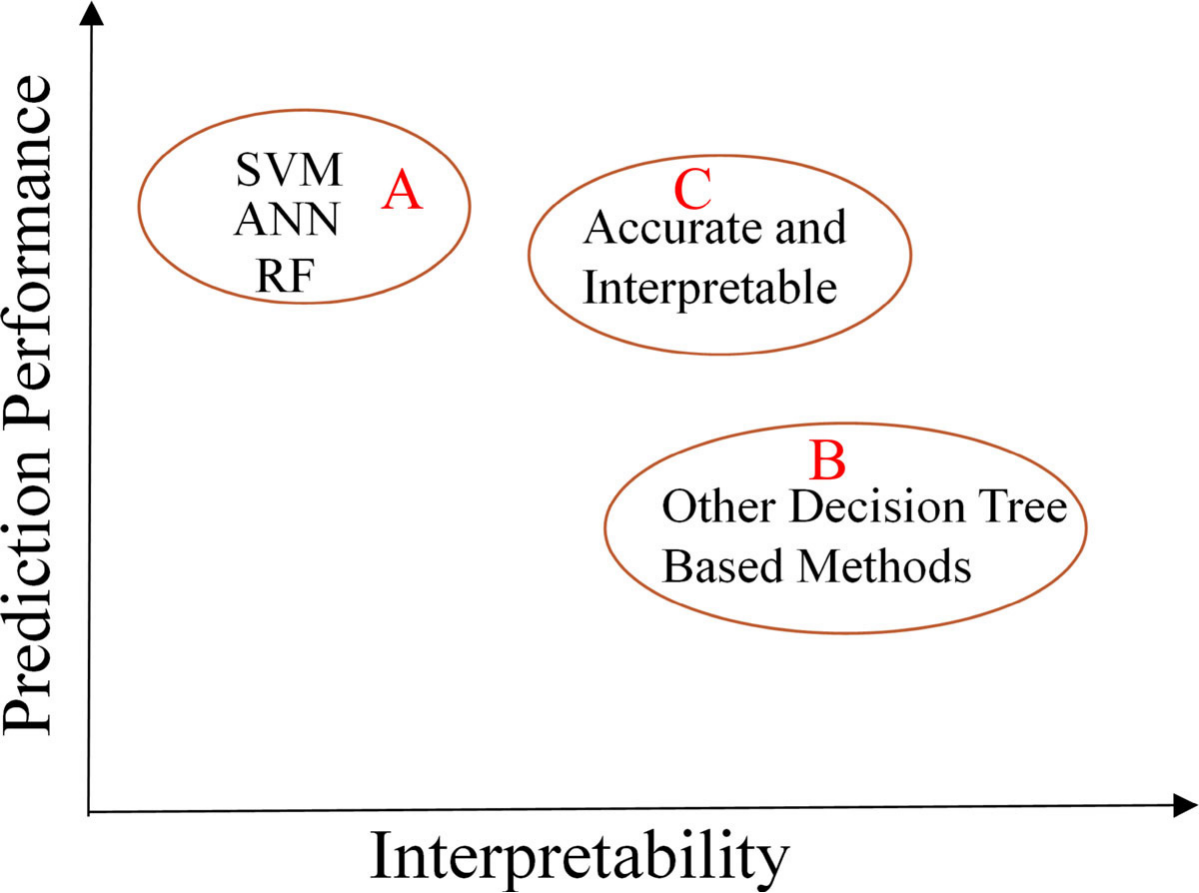
**Interpretability versus prediction performance**. Algorithms that fall into region C have relatively balanced prediction performance and interpretability. SVM: Support Vector Machines, ANN: Artificial Neural Network, RF: Random Forests.

In many real applications, it is vital to have an interpretable model (e.g., relevant features and predictive rules) and high performance prediction at the same time to understand the underlying problem well. Some of the state-of-the-art algorithms like Support Vector Machines (SVM) [1], Artificial Neural Network (ANN) [2], and Random Forests (RF) [3], generally predict the outcome with high accuracy. But other than accuracy, it is hard to interpret the models built since they either are "black box" model, or include so many decision rules that human cannot explain them clearly. On the other hand, some algorithms, especially those based on decision trees, are easy to interpret. However, the prediction performance is usually low compared to SVM, ANN, or RF. See Figure 1 for an illustration regarding the interpretability-prediction performance space. Basically, to help explain the generated model, it is desirable to have an algorithm that falls on the region C. Finding a right tradeoff between prediction performance and model interpretability is thus important.

Decision trees use a tree structure to represent a partition of the space. From the root node to each leaf node of a decision tree, we can consider it as a decision rule. Decision rule based algorithms are well known for their capability of shedding light on the decision process in addition to making a prediction. Another factor affecting the interpretation of model generated from data is feature selection. In general, fewer features involved in the model will make it less complex and more interpretable. There is a rich resource of prior work on rule-based learning and feature selection in the fields of bioinformatics and statistical learning. It is beyond the scope of this article to supply a complete survey of the respective areas. Below we review some of the main findings most closely related to this article.

### Our contribution

In many biological problems, building a good predictive model that explains the problem well is the ultimate goal of modelling.

High performance and concise representation (i.e., a small rule set and a small feature set) are two important requirements of rule learning methods. Regression tree based methods usually generate a small set of rules. However, their performance is relatively low compared with those using regression with SVM (Support Vector Regression) and random forests. An RF generally has high performance, but generates a large number of rules. It is difficult to interpret the model using a large number of rules. In this article, we take an iterative approach to regularize random forests to obtain refined rules without compromising the performance. RF has an ensemble of regression trees and covers more candidate rules compared with a single decision tree. Regularization keeps only a small number of rules that are the most discriminative. We take an embedded approach with a greedy backward elimination strategy for feature elimination.

We combine rule extraction and feature elimination method iteratively. The result of rule extraction is used for feature elimination. The selected features are then fed into RF and there is 1-norm regularization step to extract important rules. The iterative alternating approach continues until the selected subset of features does not change. Only a few rule learning algorithms are geared toward regression problems as opposed to classification problems. In addition to application to classification case, we apply this iterative approach to another category of learning algorithm - regression rule learning, extending its domain of usage.

It is important to evaluate the quality of the algorithm in terms of prediction performance and interpretability. We use a set of metric to evaluate rule quality as follows:

1 Accuracy: *R*^2^.

2 Variance of accuracy.

3 Interpretability: number of rules.

4 Interpretability: number of variables used in rule.

5 Robustness to noise.

## Methods

In this section, we describe the proposed method. First, we present an approach to find the \right" trade off between prediction performance and model complexity using regularization. We then describe our approach by showing a mapping of the forest generated by RF to rule space where many of rules are being removed by 1-norm regularization. Then we present several metrics for evaluation of accuracy and interpretability respectively.

### Balancing accuracy and model complexity with regularization

Machine learning algorithms normally learn a function *f *from input *x *to output *y*, that is,

y=f(x).

A loss function *L*(*x, y, f*) is minimized with respect to *x, y*, and *f*. The loss function usually takes the form of error penalty, for example, the squared error:

$$L\left(x,\;y,\;f\right)={\left(y\;-f\left(x\right)\right)}^{2}$$

which aims at achieving low error rate on training data. It is common that model constructed this way works very well on training data, but not on test data. This is called overfitting. To avoid the overfitting problem, we can add a complexity penalty to the loss function, for example, *L*_1 _regularization:

$${\left(y-f\left(x\right)\right)}^{2}+\lambda ∥w∥{}_{1}$$

where *w *is parameter in the model, *λ *is tuning parameter to balancing the accuracy and complexity. It can generates relatively less complex model comparing with the previous one. In this article, we use 1-norm regularization. Due to the sparse solution of 1-norm regularization, the model constructed above is much simplified. 1-norm regularization has been widely applied in statistics and machine learning, e.g., [4,5], and [6]. The above optimization can be solved by a linear program solver (LP).

### Rule elimination using 1-norm regularization from random forests mapped rules

From training samples, we can construct a random forest. As the path from a root node to a leaf node in a decision tree is interpreted as a regression rule, a random forests is equivalently represented as a collection of regression rules. Because each sample traverses each tree from root node to one and only one leaf node, we define a feature vector to capture the leaf node structure of a RF. For sample **^x^***i*^, ^the corresponding feature vector that encodes the leaf node assignment is defined as **X***^i ^*= [*X*_1_*^, . . . , ^X^q^*]*^T ^*where *q *is the total number of leaf nodes in the forest,

(1) $$X_{i}=\left\{\begin{array}{cc} a_{j} & \text{if}\;{\text{x}}_{i}\;\text{reaches}\;\text{the}\;j\text{ - th}\;\text{leaf}\;\text{node},\\ 0 & \text{otherwise}. \end{array}\right)$$

i=1,⋯,l,j=1,⋯,q.

where *a_j _*is the target value at leaf node *j*. We call the space of **X***_i_*^'^s the rule space. Each dimension of the rule space is defined by one regression rule. The above mapping is an extension of binary mapping applied in [7,8] to the regression case.

Using the above mapping, we obtain a new set of training samples in the rule space,

$$\left\{\left({\text{X}}_{1},\;y_{1}\right),\;\left({\text{X}}_{2},\;y_{2}\right),\;\cdots \;,\;\left({\text{X}}_{l},\;y_{l}\right)\right\}.$$

In rule space, we consider the following form

(2) $$y=w^{T}\text{X}+b.$$

where weight vector **w **and scalar *b *define linear regression function for the sample. The weights in (2) measure the importance of rules: the magnitude of a weight indicates the importance of the rule. Clearly, a rule can be removed safely if its weight is 0. Rule elimination is therefore formulated as a problem of learning the weight vectors.

We use the technique described in previous section, consider the following learning problem using 1-norm regularization:

(3) $$\begin{array}{c} \underset{\text{w},\xi_{i}}{\text{min}}\;\left(\lambda ||\text{w}{||}_{1}+\overset{l}{\underset{i=1}{\sum }}\xi_{i}\right)\\ \text{s}.\text{t}.\;\;|{\text{w}}^{T}{\text{X}}_{i}+b-y_{i}|\leq \xi_{i}\\ \;\;\;\xi_{i}\geq 0,\;i=1,\;\cdots \;,\;l. \end{array}$$

**Figure 2:**
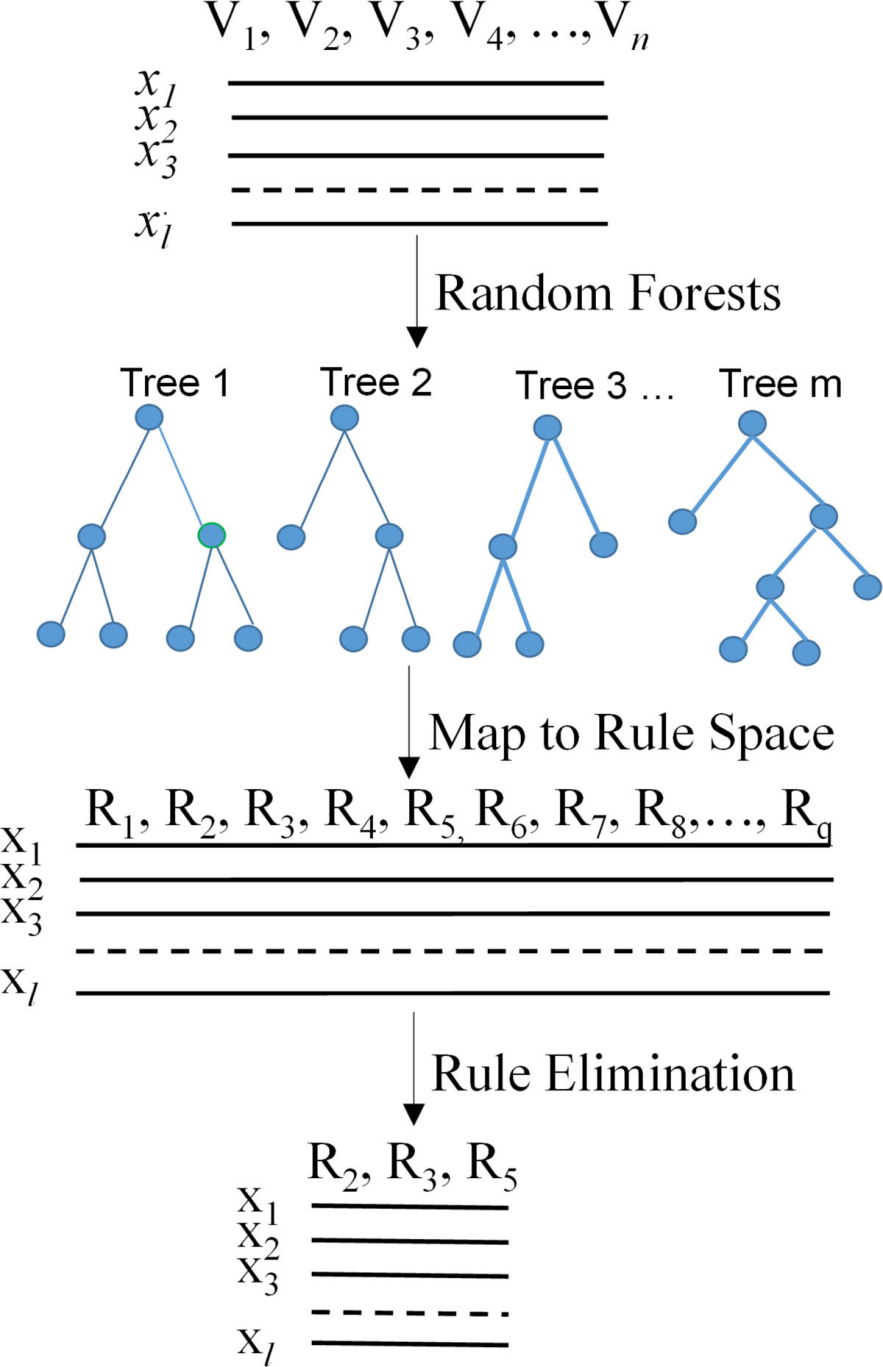
**Mapping samples to rule space and eliminate rules**. Random forests model is generated from training samples. Viewing a path from root node and a leaf node of a decision tree as a decision rule, we can encode data into rule space. Some of rules can be eliminated without affecting the prediction performance.

The solution to the above optimization problem is usually sparse, controlled by regularization parameter *λ. λ *is chosen by cross validation on the training set. Rules with zero weights *w *can be removed. Figure 2 illustrate the process shown in this section.

### Combined rule extraction and feature elimination

It is assumed that only important features are kept in the remaining rule. Features that do not appear in the rules extracted using (3) are removed because they have no or little effect on the regression problem. In this way, we can select rules and features together.

It is possible to further select rules from a RF built on the selected features to get a more compact set of rules. This motivates an iterative approach. Features selected in the previous iteration are used for constructing a new RF. A new set of rules is then extracted from the new RF. This process continues until the selected features do not change.

**Figure 3:**
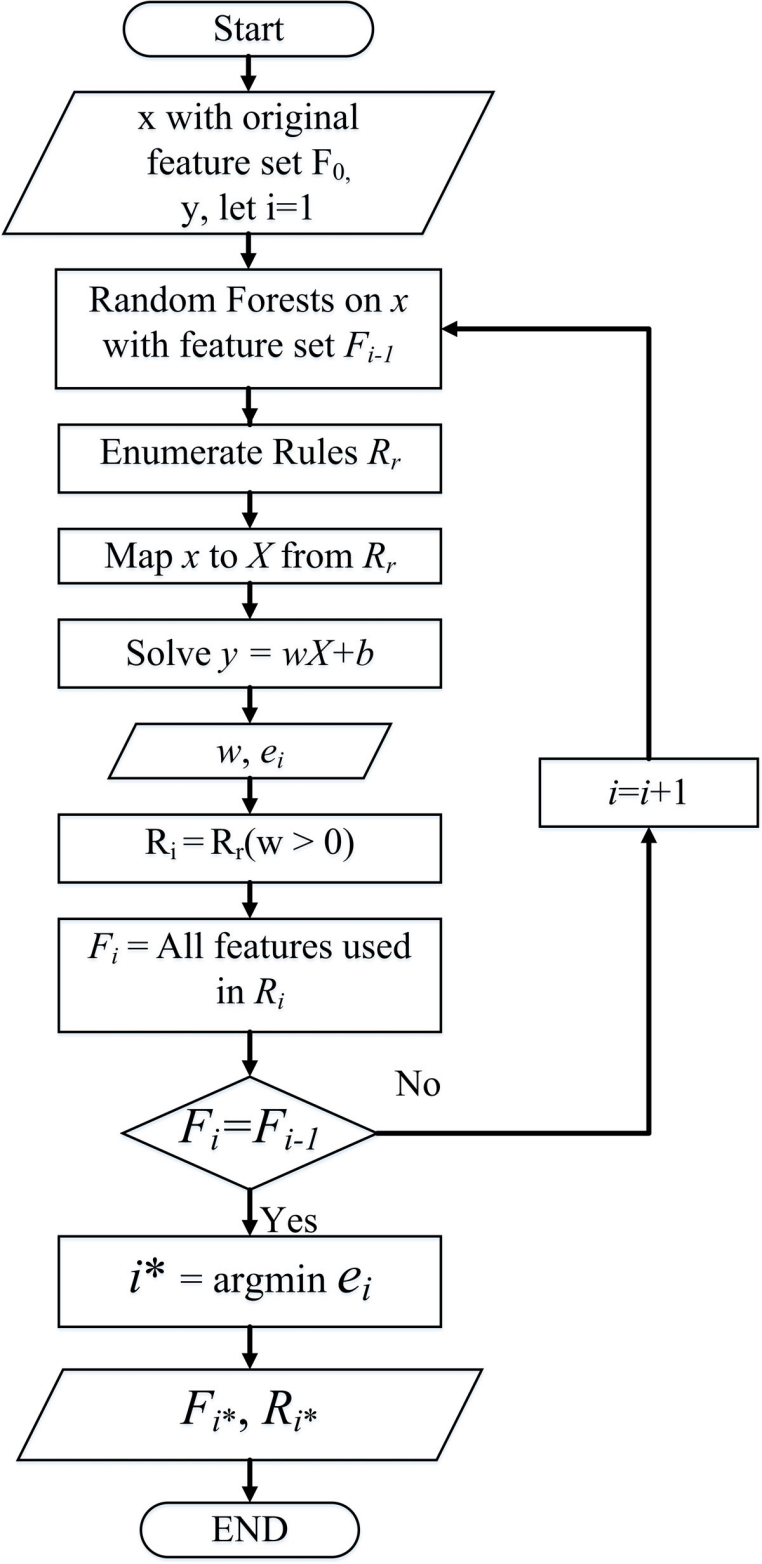
**Flowchart of the algorithm**. The whole process contains an iterative rule generation and elimination as well as feature elimination until there is no feature eliminated.

Figure 3 illustrate the overall workflow of the algorithm.

### Evaluation of results

R squared [9] statistics measures the goodness of fit of the models to the data. It is used to describe how well the predictions fit on test data. Let *y*^′ ^denote prediction values from the algorithm, $\bar{y}$ denote mean value of target variable, the formulation of R squared is:

(4) $$R^{2}=1-\frac{SS_{err}}{SS_{tot}}$$

where $SS_{err}=\sum {\left(y_{i}-y^{\prime }\right)}^{2},\;SS_{tot}=\sum {\left(y_{i}-\bar{y}\right)}^{2},\;i=1,\;\ldots ,\;n,\;n$ is number of test samples. An R squared value closer to one indicates better performance. A simple evaluation on the quality of a regression algorithm is the standard deviation of R squared based on multiple runs. For the interpretability, a small set of rules and concise rules are naturally easier for human to interpret.

In general, random forests classification is more robust against noise compared with many other methods [10]. There is a limited research, however, on whether random forests regression based methods are also robust. One straightforward method is to introduce some noise into the data and then compare the difference between R squared with and without noise. The smaller the difference is, the more robust the algorithm is to noise.

## Results and discussion

### Datasets

In this section, we first describe the data sets used. We then present detailed results and discussion.

**Figure 4:**
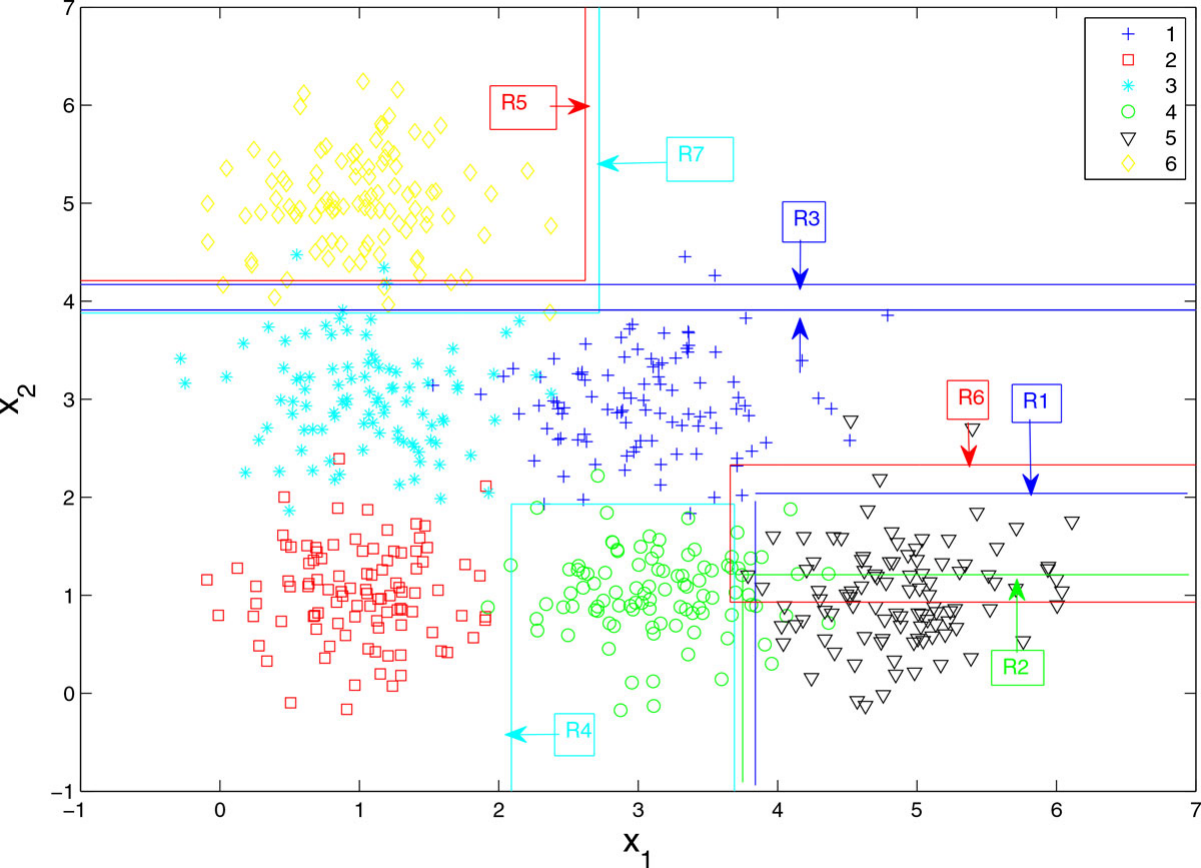
**Illustration on artificial data set**. Artificial data set described by dots and regression result demonstrated by partition and target prediction in text box.

We first test our method on an artificial data set. The data is illustrated in Figure 4. The target values are 1 through 6 corresponding to different shapes and colors. For each target value, 100 samples are generated according to different mean values with standard deviation of 0.5. The relationship between target variable and input variable X1 and X2 is nonlinear.

We then applied our method to several data sets from real applications summarized in Table 1. The first data set is Stockori flowering time data set [11]. The flowering time of 697 plants were collected. The prediction of flowering time is based on 149 genotypes of the plants.

**Table 1:** Some statistics of data sets.

Data	Number of Samples	Number of Features
Stockori Floweringtime	697	149
Parkinsons Telemonitoring	5875	19
Breast Cancer Wisconsin (Prognostic)	198	32
Relative location of computed tomography (CT) slices on axial axis	2140	384
Seacoast	2250	16
TCGA Glioblastoma multiforme	427	12042

The Parkinson's Telemonitoring data set [12] contains biomedical voice measurement from 42 people with early-stage Parkinson's disease. There are 5875 total voice recordings. The goal is to predict total Uni-fied Parkinson's Disease Rating Scale (UPDRS) scores from the voice measures and other features of patients. Breast Cancer Wisconsin (Prognostic) data set [13] is constructed using a digitized image of a fine needle aspirate (FNA) of a breast mass from breast cancer patients. Characteristic features are computed from the images. The prediction is the recurrence time or disease-free time after treatment. The Relative location of computed tomography (CT) slices on axial axis data set [14] consists of 384 features extracted from CT images. These features are derived from two histograms in polar space. The response variable is relative location of an image on the axial axis ranging from 0 to 180 where 0 denotes the top of the head and 180 the soles of the feet. We randomly choose 2140 CT images for the analysis. The above three data sets are retrieved from University of California, Irvine (UCI) repository [15].

The Seacoast data set is a collection of sensor readings about different biochemical concentrations under various humidity and temperatures. Concentrations of the biochemical can be inferred from sensor responses using our approach. The data set is pre-processed by normalizing raw sensor responses, calibrating sensor data according to standard no biochemical input conditions and according to the time delay in the sensor response, if available. Humidity levels and temperatures are also factored out first by using a regression based approach. This results in sensor responses being in the same scale. 2250 times points are sampled and used.

The TCGA Glioblastoma multiforme (GBM) data is downloaded from The Cancel Genome Atlas (TCGA) data portal (https://tcga-data.nci.nih.gov/tcga/). 548 gene expression profiles were retrieved from the Broad Institute HT HG-U133A platform (Affymetrix, Santa Clara, CA, USA). Each gene expression profile consists of normalized expression data of 12042 genes. The survival information of patients is retrieved from TCGA clinical data. After removing gene expression samples with unknown survival information, 427 samples were used in our analysis.

### Results on artificial data set

Random forests regression generates 11700 rules with *R*^2 ^of 0.87. Our method gets 7 rules with *R*^2 ^of 0.66. There is not too much loss in the prediction performance. The predicted rules are as follows:

1 **IF ***x*_2 _≤ 2.04 and *x*_1 _*>*3.84 **THEN ***y *= 5

2 **IF ***x*_2 _≤ 1.21 and *x*_1 _*>*3.75 **THEN ***y *= 5

3 **IF ***x*_2 _≤ 4.17 and *x*_2 _*>*3.91 **THEN ***y *= 6

4 **IF ***x*_1 _≤ 3.68 and *x*_2 _≤ 1.93 and *x*_1 _*>*2.09 **THEN ***y *= 4

5 **IF ***x*_2 _*>*4.21 and *x*_1 _≤ 2.62 **THEN ***y *= 6

6 **IF ***x*_2 _≤ 2.33 and *x*_2 _*>*0.93 and *x*_1 _*>*3.66 **THEN ***y *= 5

7 **IF ***x*_2 _*>*3.88 and *x*_1 _*<*2.72 **THEN ***y *= 6.

They are also illustrated in Figure 4. Numbers in text boxes are prediction values of target variable. Lines generated from rules partition the original space. Many of these rules align well with the partition. Noted that multiple run of our approach generates different sets of rules. The number of extracted rules also changes. The partitions in those rules align well with the partition also.

### Results on different data sets

The following tables present the result of our proposed methods on different data sets. Results are from test data.

From Table 2, we can see that in all data sets, the number of rules is reduced significantly comparing to random forests yielding less than 1% of the original number of rules in the forest. At the same time, the performance measured by *R*^2 ^does not change too much. In most data sets, except Parkinson's Telemonitoring data set, RF gives the best performance. Support vector regression is the least competitive in the cases we tested. Our approach stands somewhere in the middle. Note that on Stockori flowering time data set, the target variable, flowering time, is ordered. Here we simply treat it as numbers. The performance is comparable with RF. In Breast Cancer Wisconsin (Prognostic) data set, the predictive performance is low indicating it is a hard problem. Our approach does not work well on this data set either. It may be resulted from over pruning the rules.

**Table 2:** Results on different data sets.

**Numbers after *± *are standard deviation. SVR is support vector regression**.			
	**Random Forests**	**Our Approach**	**SVR**

Stockori Flowing Time			

*R*^2^	0.54 *± *0.00	0.45 *± *0.05	0.28 *± *0.03

Number of Rules Selected	66020 *± *187	348 *± *33	NA

Number of Features Used in a Rule	8.8 *± *1.9	7.5*± *1.74	NA

Number of Features Selected	149 *± *0	135 *± *31	149 *± *0

Parkinson's Telemonitoring			

*R*^2^	0.15 *± *0.02	0.06 *± *0.02	0.17 *± *0.02

Number of Rules Selected	644789 *± *414	3796 *± *0	NA

Number of Features Used in a Rule	9.72*± *2.14	7.4 *± *1.86	NA

Number of Features Selected	19 *± *0	19 *± *0	19 *± *0

Breast Cancer Wisconsin (Prognostic)			

*R*^2^	0.04 *± *0.02	-0.19 *± *0.16	-0.04 *± *0.04

Number of Rules Selected	43907 *± *58	126 *± *2	NA

Number of Features Used in a Rule	7 *± *3	3 *± *1.49	NA

Number of Features Selected	32 *± *0	31 *± *1	32 *± *0

Relative location of CT slices on axial axis			

*R*^2^	0.92 *± *0.01	0.77 *± *0.09	0.26 *± *0.00

Number of Rules Selected	172984 *± *143	901 *± *15	NA

Number of Features Used in a Rule	12 *± *3.12	8 *± *2.53	NA

Number of Features Selected	384 *± *0	20 *±± *5	384 *± *0

Seacoast			

*R*^2^	0.64 *± *0.02	0.59 *± *0.10	-0.19 *± *0.00

Number of Rules Selected	120771 *± *161	385 ≤ 5	NA

Number of Features Used in a Rule	14 *±± *3	6 *± *1.91	NA

Number of Features Selected	16 *± *0	16 *± *0	16 *± *0

TCGA Glioblastoma multiforme			

*R*^2^	0.04 *± *0.01	-1.94 *± *0.67	-0.09 *± *0.00

Number of Rules Selected	53539 *± *31344	279 *± *6	NA

Number of Features Used in a Rule	3 *± *2	2 *± *1	NA

Number of Features Selected	12042 *± *0	2 *± *1	12042 *± *0

The standard deviation on the *R*^2^, number of rules selected, number of features selected demonstrates that the methods are stable on most of these data sets. The standard deviation of *R*^2 ^is obtained from the average of *R*^2 ^over ten runs.

One example rule set from top five rules based on absolute value of weight in Breast Cancer Wisconsin (Prognostic) data set are as follows:

1 **IF ***v*_22 _*>*30.27 and *v*_1 _*≤ *17.23 and *v*_5 _*>*0.09 and *v*_11 _*>*0.24 and *v*_25 _*≤ *0.16 and *v*_14 _*>*28.38 and *v*_20 _*>*0.00 and *v*_20 _*≤ *0.01 **THEN ***y *= 64.5

2 **IF ***v*_12 _*≤ *1.17 and *v*_9 _*≤ *0.18 and *v*_3 _*>*88.13 and *v*_16 _*>*0.02 and *v*_21 _*>*23.37 **THEN ***y *= 57

3 **IF ***v*_4 _*>*814.40 and *v*_12 _*≤ *0.70 **THEN ***y *= 101.33

4 **IF ***v*_17 _*≤ *0.05 and *v*_30 _*≤ *0.10 and *v*_19 _*≤ *0.01 and *v*_31 _*>*0.70 and *v*_16 _*≤ *0.03 and *v*_23 _*≤ *130.75 **THEN ***y *= 69.25

5 **IF ***v*_23 _*>*123.70 and *v*_29 _*>*0.26 and *v*_2 _*≤ *18.43 and *v*_17 _*>*0.03 **THEN ***y *= 109.2.

where *v*_1 _is mean radius, *v*_2 _is mean texture, *v*_3 _is mean perimeter, *v*_4 _is mean area, *v*_5 _is mean smoothness, *v*_9 _is mean symmetry, *v*_11 _radius standard error (SE), *v*_12 _is texture SE, *v*_14 _is area SE, *v*_16 _is compactness SE, *v*_17 _is concavity SE, *v*_19 _is symmetry SE, *v*_20 _is fractal dimension SE, *v*_21 _is worst radius, *v*_22 _is worst texture, *v*_23 _is worst perimeter, *v*_25 _is worst smoothness, *v*_29 _is worst symmetry, *v*_30 _is worst fractal dimension, and *v*_31 _is tumor size. Among these rules, size, shape, and texture features occur more often than other features indicating these features are more important than other features in deciding breast cancer. This result is similar to conclusion made in [16] and [17].

**Figure 5:**
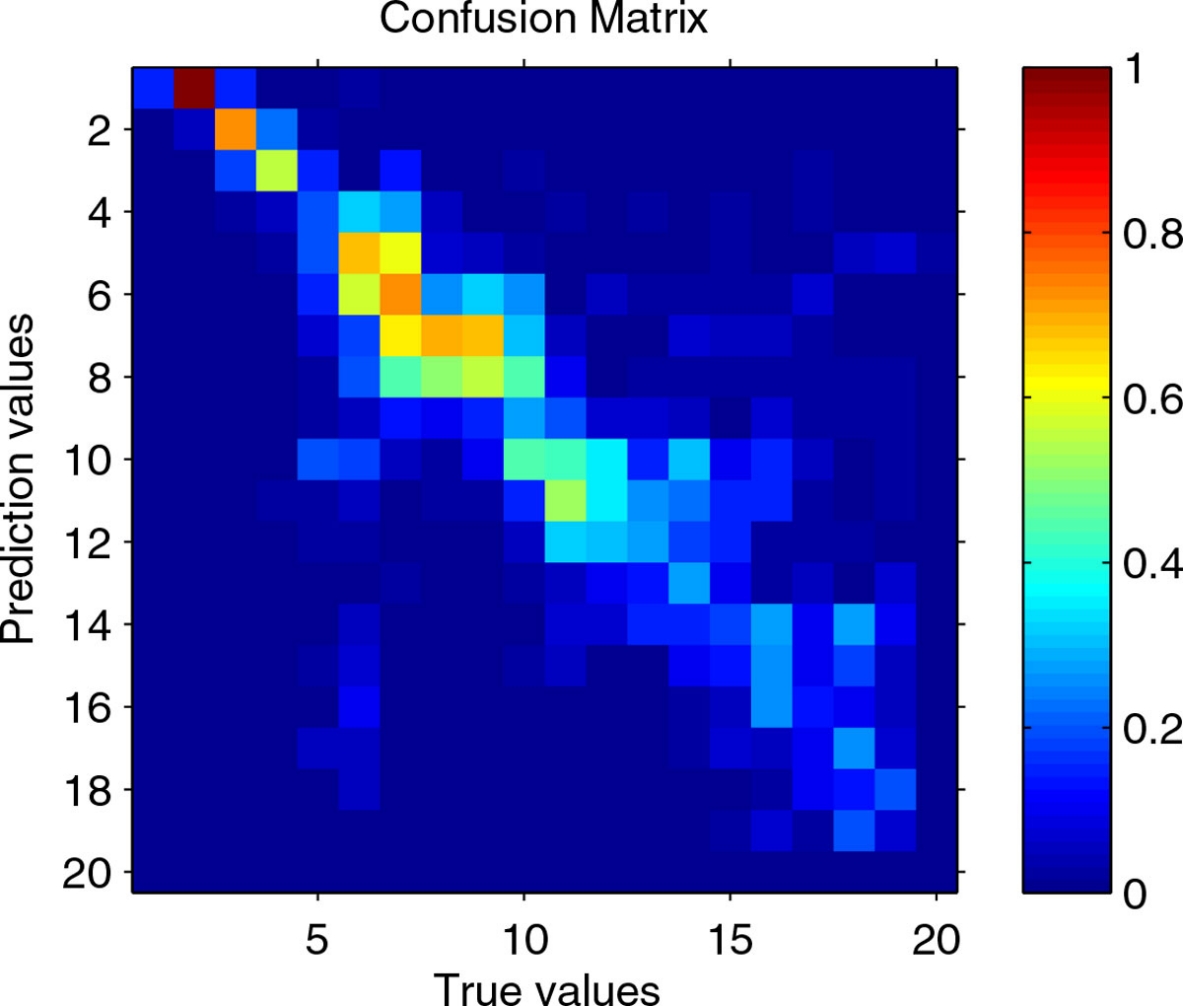
**Heatmap of confusion matrix**. Target values are partitioned into 20 intervals. True values and prediction values are then discretized into those intervals. Confusion matrix between true values and prediction values is formed by counting the number of matches within each interval. The counts are divided by the maximum count.

To illustrate how prediction values matched true values, we use an approach similar to [18], which was used for clustering analysis. Here we partition the target values in different intervals, and then count how many samples fall into the same interval for both prediction and true values. The resulting confusion matrix can be visualized to get an idea how they match. Figure 5 shows that most of the sample matches are in the diagonal of the matrix which indicate correct match.

**Figure 6:**
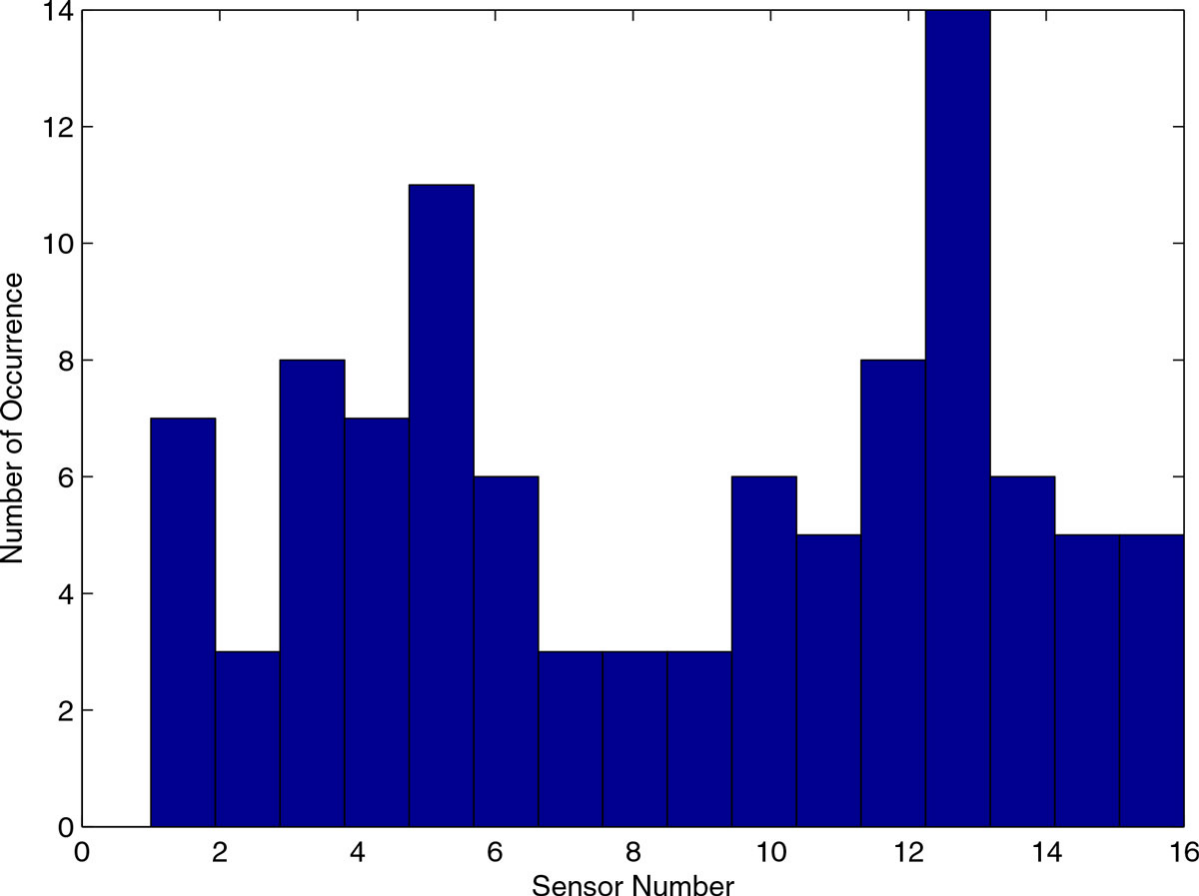
**Number of occurrence of sensors in top ten rules**. Top ten rules are extracted according to absolute value of weights. The number of occurrence of each sensor is enumerated.

Top ten rules are extracted from Seacoast data based on absolute value of weight of rules. Figure 6 shows the number of occurrence of sensors in top ten rules. The sensors are numbered from 1 to 16 accordingly: C0 = 0 MSS556 cp29I Ethyl Cellulose, C1 = 1 MSS556 Ethyl Cellulose, C2 = 2 MSS556 2STH162 (HC), C3 = 3 MSS556 2STH162 (HC), C4 = 4 MSS556 PECH, C5 = 5 MSS556 PECH, C6 = 6 MSS556 PEVA 40%, C7 = 7 MSS556 PEVA 40%, C0 = 0 MSS557 cp27i Ethyl Cellulose, C1 = 1 MSS557 Ethyl Cellulose, C2 = 2 MSS557 2STH162 (HC), C3 = 3 MSS557 2STH162 (HC), C4 = 4 MSS557 PECH, C5 = 5 MSS557 PECH, C6 = 6 MSS557 PEVA 40%, C7 = 7 MSS557 PEVA 40%. From Figure 6, two sensors, C5 = 5 MSS556 PECH and C5 = 5 MSS557 PECH, used more often, suggesting it is more important or effective in determining chemical concentration.

**Figure 7:**
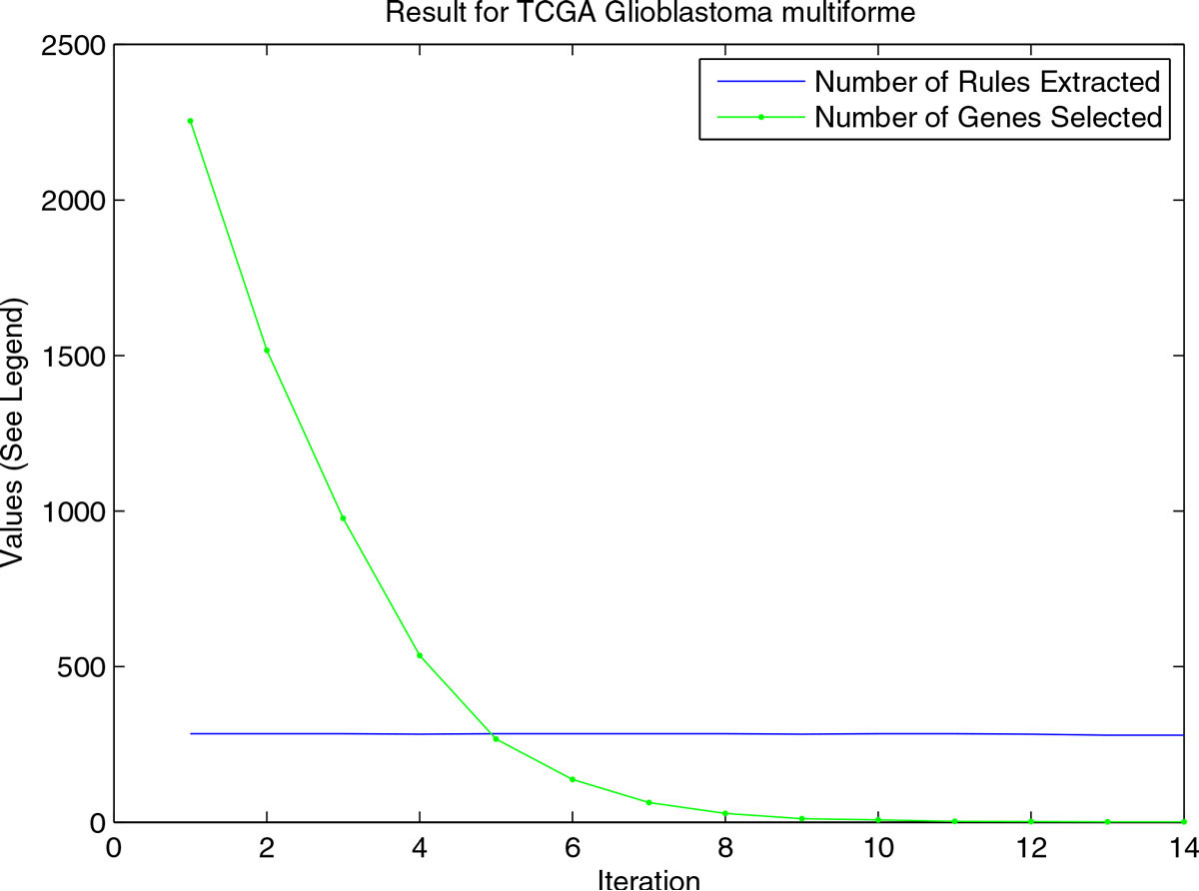
**Dynamics of number of rules and genes during iteration**. Number of rules extracted and number of genes selected during the iterations of our approach.

For TCGA Glioblastoma multiforme data set, we can see an interesting result that the number of genes is reduced during iteration, while the number of remaining rules is almost constant after the first iteration. The prediction performance are not good for any of the three algorithms indicating it is a harder problem, and current gene expression profiles may not provide the necessary information for the survival prediction. See Figure 7.

### Results with noisy data

To illustrate the robustness of our approach on noisy data, we randomly add some Gaussian noise in the Stockori flowering time data set with probability 0.3 for each feature in a sample. Storckori flowering time was randomly sampled for training and testing sets. The sets were used for the experiments. 10 runs were done for each method. The mean of Gaussian noise is 0.5, while its standard deviation is 0.2. From Table 3, we can see that support vector regression has the highest *p *value on paired t test on difference in mean *R*^2^s of SVR on data without noise and data with noise, it was not affected too much by Gaussian noise. But its *R*^2 ^is still the lowest among all three methods. The *p *value shows that there is no statistical significance between results with noise and without noise. Our approach has similar values of *R*^2 ^compared with those of random forests. Increasing the probability of noise from 0.3 to 1, both random forests and the proposed approach are affected by the increased noise level.

**Table 3:** Result on stockori flowering time data set with noise.

**Numbers after *± *are standard deviation. SVR is support vector regression**.			
	**Random Forests**	**Our Approach**	**SVR**

*R*^2^	0.43 *±*0.02	0.36 *± *0.07	0.24 *± *0.05

D	0.13	0.1	0.01

## Conclusion

We propose to use an ensemble of decision rules generated from random forests and 1-norm regularization to balance prediction performance and interpretability of regression problems. The method selects a small number of rules (using a small number of features) while retaining performance comparable to RF, better than SVR in most cases.

Due to decision trees' ability handling mixed data type, our approach is able to handles data with mixed type.

We also study robustness of our approach in the presence of noise. The prediction performance is still comparable with random forests in terms of performance within small amount of Gaussian noise.

Regression problems are generally harder than classification problems both in terms of prediction performance and interpretability [8]. Therefore, care should be taken when interpreting the results.

## Competing interests

The authors declare that they have no competing interests.

## Authors' contributions

YC and DW provided guidance and planning for the project. SL produced the program, ran the experiment, and wrote the manuscript. SD, SP, and TM contributed in preparing data and discussions. XD participated in design of experiment. All authors read and approved the final manuscript.

## Declarations

The full funding for the publication fee came from Mississippi Experimental Program to Stimulate Competitive Research (EPSCoR).

This article has been published as part of *BMC Systems Biology *Volume 8 Supplement 3, 2014: IEEE International Conference on Bioinformatics and Biomedicine (BIBM 2013): Systems Biology Approaches to Biomedicine. The full contents of the supplement are available online at http://www.biomedcentral.com/bmcsystbiol/supplements/8/S3.
